# Modeling erythrocyte electrodeformation in response to amplitude modulated electric waveforms

**DOI:** 10.1038/s41598-018-28503-w

**Published:** 2018-07-05

**Authors:** Yuhao Qiang, Jia Liu, Fan Yang, Darryl Dieujuste, E. Du

**Affiliations:** 1 Department of Ocean and Mechanical Engineering, Florida Atlantic University, Boca Raton, FL 33431 USA; 2 School of Mechanical Engineering, Nanjing University of Science and Technology, Nanjing, 210094 China; 3 Department of Electrical Engineering, Florida Atlantic University, Boca Raton, FL 33431 USA

## Abstract

We present a comprehensive theoretical-experimental framework for quantitative, high-throughput study of cell biomechanics. An improved electrodeformation method has been developed by combing dielectrophoresis and amplitude shift keying, a form of amplitude modulation. This method offers a potential to fully control the magnitude and rate of deformation in cell membranes. In healthy human red blood cells, nonlinear viscoelasticity of cell membranes is obtained through variable amplitude load testing. A mathematical model to predict cellular deformations is validated using the experimental results of healthy human red blood cells subjected to various types of loading. These results demonstrate new capabilities of the electrodeformation technique and the validated mathematical model to explore the effects of different loading configurations on the cellular mechanical behavior. This gives it more advantages over existing methods and can be further developed to study the effects of strain rate and loading waveform on the mechanical properties of biological cells in health and disease.

## Introduction

Red blood cell (RBC, or erythrocyte) plays an important role to transport oxygen to tissues and organs and carry carbon dioxide back to lungs. Its unique biconcave disc shape, the viscoelastic properties of the membrane, and the viscosity of the cytoplasm are the primary factors that affect the ability of a RBC to pass through the narrow capillaries and the inter-endothelial slits in human spleen^1,2^. During blood circulation through the cardiovascular system, RBCs are subjected to dynamic and cyclic loading of fluctuating stresses and strains when traversing arteries, capillaries, and veins of different kinematic parameters^3^. Studying biomechanical properties of single RBCs has allowed us to better understand and accurately model various physiological processes, such as blood rheology^4^, splenic filtration^5^, discocyte-echinocyte morphological transformation^6^, hemolysis and fragmentation^7^, cell adhesion^8^, and cell-cell interactions^9^. Viscoelasticity of RBC membranes is of particular interest for the studies of blood diseases^10^. Membrane biomechanics can potentially serve as biomarkers of various blood diseases, such as malaria^11^ and sickle cell anemia^12^.

Various experimental strategies for single-cell biomechanics characterization, such as micropipette aspiration^13^, optical tweezers^14^, and magnetic twisting^10^, are widely used and established. These tools often have limited throughput. In the latter two approaches, application of force needed to deform cells requires pre-attachment of microbeads to cell membranes, leading to additional time for sample preparation. Label-free methods could overcome these limitations. Microfluidics has been recognized as a potential tool to develop experimental models^15^ for the biomechanical studies of living cells, complementary to those classical physics and engineering techniques. These experimental approaches can be found in a comprehensive review^16^.

Passive shear flow^8^ and dielectrophoresis (DEP)^17–19^ are the two main mechanisms implemented in microfluidics for characterizing cell biomechanics and blood rheology. DEP force from Maxwell-Wagner type polarization^20^ exerted on a cell is along with the field lines and its magnitude is determined by the relative polarizability of the cell and the surrounding medium, electrical field strength, cell shape and size^21^. This effect has been utilized to study electrodeformation of single cells, such as RBCs^22,23^, mammalian cells^24^, plant protoplasts^25^, and cervical cancer cells^26^.

Most of the existing work on DEP force calibration is based on approximated mathematical models, such as effective dipole moment (EDM) method and spherical cell model^27^. These approximations are reasonably accurate when cells are not in proximity of electrodes. Improved DEP mathematical models have been developed to consider the multi-layer composition and ellipsoidal structure of deformed cells^17,28^. For cells with comparable size to the electrodes or when cells move to the vicinity of the electrodes, cellular influence on the distribution of electrical field is no longer negligible. In such case, Maxwell stress tensor (MST) method can be used to calculate the electrical forces exerted on deformed cells with improved accuracy^28,29^. In combination with the mathematical model of membrane viscoelasticity developed by Evans and Hochmuth^30^, shear elastic modulus and viscosity of RBC membranes can be determined^18,19,31^. However, experimental analysis of cellular biomechanics by the DEP method is still limited to the investigation of the creep deformation in cell membranes by a sudden actuation and deactivation of electric excitation. Many existing DEP-based cell biomechanics studies are therefore limited to study the cell behavior under a fixed level of load, which does not provide comprehensive and detailed information of nonlinear viscoelastic behavior of cell membranes. Modulation of the magnitude of DEP forces using Amplitude Shift Keying (ASK) technique offers new opportunities to design loading configurations for the study of single-cell biomechanics^32^.

RBCs exhibit viscoelastic behavior in response to external loading. The shear stress-strain relationship of cell membranes was identified to be linear for small deformations and nonlinear for large deformations^33^. The shear modulus of membranes is dependent on both shear strain and strain rate, especially for large deformations^34,35^. A classical model to explain the viscoelastic behavior of the cell membranes was developed as an analogue of the classical Kelvin-Voigt solid model^36^. Other models, such as the three-stage elastic moduli^14,34^ and with an additional dual-viscosity dash pots^18^ were used to describe the nonlinear elastic and viscoelastic behaviors of RBC membranes. For healthy RBCs, the reported values of membrane shear modulus were about 2.4–13.3 µN/m^37,38^ and viscosity 0.3 to 2.8 (µN/m)·s^38^. A recent study showed that magnitude of the RBC membrane shear modulus increases with strain rate; at a shear rate of 2 ms^−1^, the value of the nonlinear shear modulus is up to 60 µN/m^39^. The nonlinear shear elasticity and viscosity of healthy RBCs were found to be dependent on the deformation ratio of the cells and can be well fitted with an exponential function for large deformations^40^.

In this paper, we develop a new experimental method to generate specific loading waveforms, such as sinusoidal loading with a variable strain rate and triangular loading with a fixed strain rate using amplitude-modulated DEP-induced electrodeformations. We examine how individually tracked RBCs respond to various DEP excitation profiles, including a sudden application/release and a gradual application/release of DEP forces in a stepwise manner, a linear manner and a sinusoidal manner, respectively. Time-variant DEP forces are calibrated based on the transient loading amplitudes and cell deformations characterized experimentally. We then evaluate and validate the mathematical model by comparing the theoretical calculations of the dynamic deformation of cell membranes to experimental observations in single RBCs subjected to a specific loading waveform at various field strengths. Furthermore, we predict the electrodeformation in a simulated cell with mean values of nonlinear viscoelasticity for a quantification of the dissipated energy in healthy RBCs.

## Results

### Stretching force calibration

**Figure 1:**
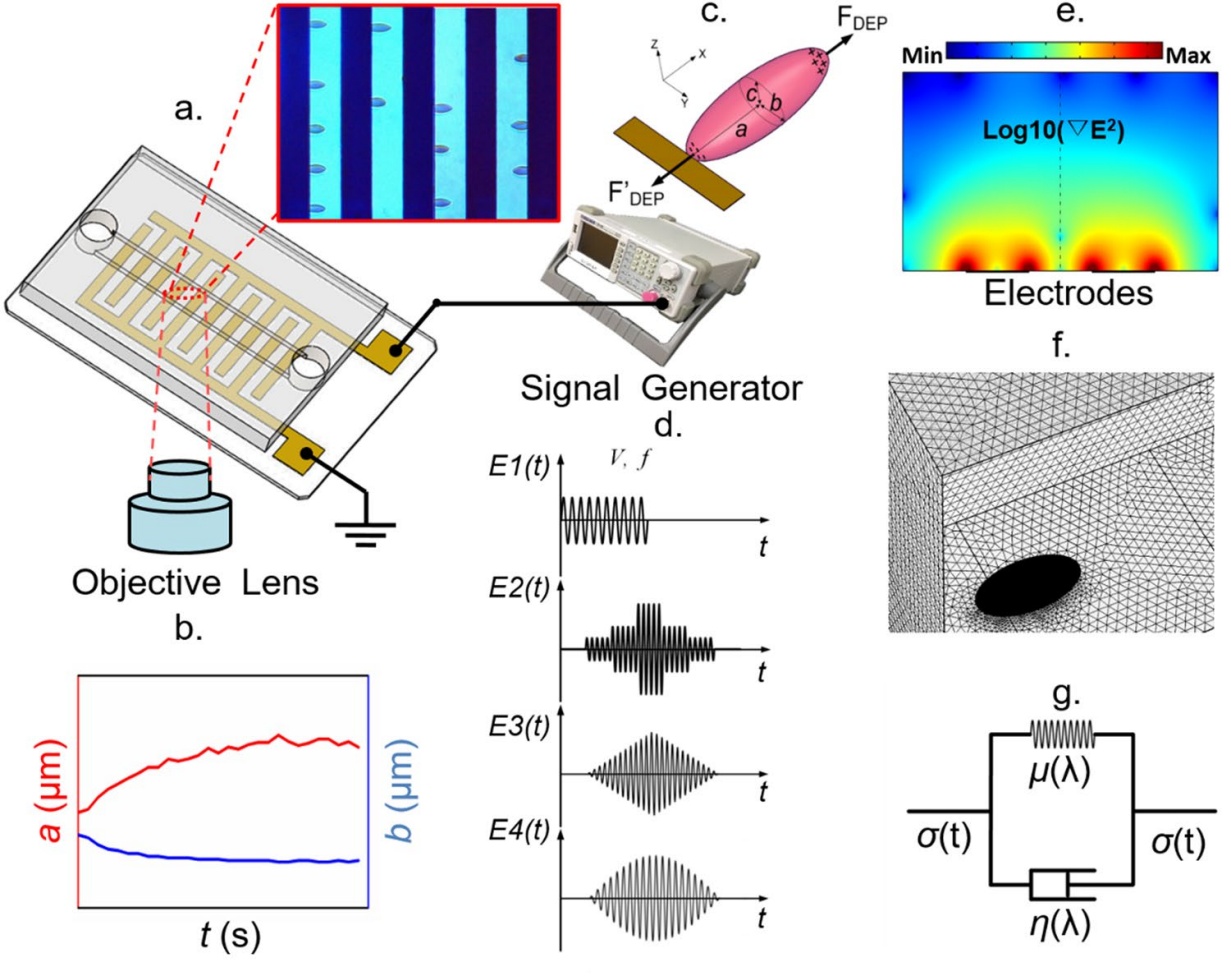
Modeling and experimental framework for biomechanical analysis of live cells using amplitude-modulated DEP in microfluidics. (**a**) Schematic of the microfluidic device with inset of microscopic view of cellular deformation. Dark strips represent the interdigitated electrode array. (**b**) Major and minor axes of ellipse fitting of a deformed cell extracted from microscopic images. (**c**) Schematic of uniaxial stretching of single RBC by positive DEP force. (**d**) Representative ASK-modulated waveforms for various DEP loading profiles. (**e**) Surface plot of the gradient of field strength square in the microfluidic device. (**f**) Finite element model of cellular electrodeformation by COMSOL Multiphysics. (**g**) Kelvin-Voigt solid model of cell deformation.

**Figure 2:**
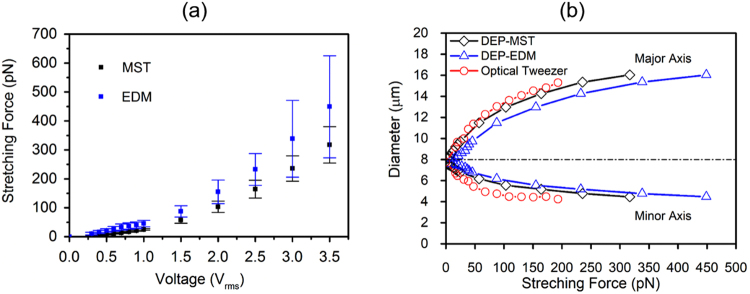
(**a**) Mean values of the DEP stretching force as a function of excitation voltage, calculated by MST and EDM methods. (**b**) Variations of the major and minor axes of RBCs against stretching force obtained by DEP technique, calibrated by MST and EDM methods, as compared to optical tweezers technique.

It was observed experimentally that single cells were stretched gradually in response to a high-frequency electrical excitation in the microfluidic device (Fig. 1a,b). Such uniaxial deformation in cell membranes, illustrated schematically in Fig. 1c, reached to a plateau where shear strain rate approached to zero due to an equilibrium between the DEP force and the membrane elastic force resultant. By increasing the voltage root mean square (V_rms_) of the modulated electrical excitations from 0.5 V_rms_ to 3 V_rms_, values of stretching forces at the corresponding equilibrium states were measured in 84 individually tracked RBCs. As described in “Methods”, stretching forces were calculated using the EDM and MST methods respectively. DEP forces calibrated by both methods have significant overlap, as illustrated in Fig. 2a. Values from the EDM method yield a higher force calibration than the MST method. The discrepancy increases with the voltage level. To facilitate comparison of the DEP technique to the standard technique, such as optical tweezers technique^34^, variations of averaged major and minor axes of healthy RBCs were plotted against the stretching force (Fig. 2b). The force-deformation relationship calibrated by the DEP-MST method was in good agreement with the optical tweezers. Therefore, MST method was selected to calibrate the DEP forces for experimental characterization of shear stresses as well as for the mathematical prediction of membrane deformation.

### Nonlinear elasticity and viscosity

**Figure 3:**
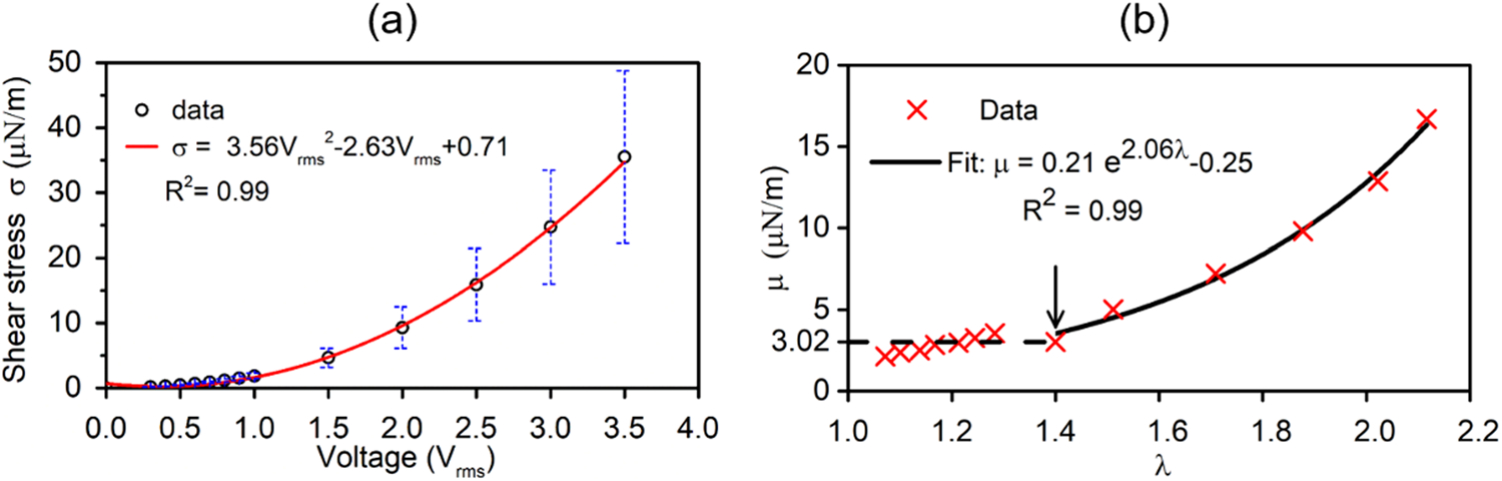
(**a**) Mean values of shear stress and a best fit function of applied voltage levels. (**b**) Mean values of membrane shear modulus and best fit functions for nonlinear elastic moduli: for small deformations (λ < 1.4), membrane shear modulus, µ = 3.02 µN/m; for large deformations (λ > 1.4), μ = 0.21 × exp(2.06 × λ) − 0.25.

Elasticity and viscosity of RBC membranes were characterized from individually tracked cells. Values of shear modulus µ and time constant t_c_ were extracted using the Kelvin-Voigt solid model. Shear stresses were determined from Eq. (6), with stretching forces calculated by the MST method. Mean values of shear stress σ were approximated as a function of V_rms_. Dependence of the membrane shear stress can be well fitted with a polynomial function of the voltage,$\,{\rm{\sigma }}=3.65\times {{{\rm{V}}}_{{\rm{rms}}}}^{2}-2.63\times {{\rm{V}}}_{{\rm{rms}}}+0.71$, as illustrated in Fig. 3a. Nonlinear relationship between membrane shear modulus μ and the extension ratio λ was characterized from the mean values of the 84 experiments and expressed as a discontinuous function (Fig. 3b). For small deformations, λ < 1.4 in present study, membrane shear modulus µ is a constant, 3.02 µN/m. For large deformations, λ > 1.4, values of µ can be well fitted with an exponential function with respect to the extension ratio, ${\rm{\mu }}=0.21\times \exp (2.06\times {\rm{\lambda }})-0.25$. Value of μ was up to 16.7 µN/m, when λ approached to 2.1. Upon a sudden release of DEP force by deactivation of the electrical excitation, stretched RBCs recovered to their stress-free state at a time-dependent rate. To evaluate the extensional recovery process, time constant t_c_ was extracted using Eq. (9). Values of t_c_ averaged from 15 individual measurements were 0.16 ± 0.04 s, 0.15 ± 0.05 s and 0.12 ± 0.02 s at voltage levels of 0.5 V_rms_, 1.0 V_rms_ and 2.0 V_rms_, respectively^40^. The overall average value of t_c_ = 0.14 ± 0.05 s was used as a constant to determine the viscosity, $\eta$ by equation ${{\rm{t}}}_{{\rm{c}}}\equiv {\rm{\eta }}/{\rm{\mu }}$.The calculated nonlinear viscosity of RBC membranes was then expressed as a function of extension ratio: when ${\rm{\lambda }}\, < \,1.4$,$\,\eta =0.42$, and when ${\rm{\lambda }} > \,1.4$,$\,\eta =0.03\times \exp (2.06\times {\rm{\lambda }})-0.035$.

### Validation of the theoretical model for DEP-induced cell deformation

Validation of the established theoretical model was performed by comparing the transient deformations of single RBCs under a switched ON/OFF load as shown by profile E1 in Fig. 1d, including an application of the high frequency electric excitation for 1 s, followed by a sudden release of the load, and a load-free state for 1 s. Three voltage levels of 0.5 V_rms_, 1.0 V_rms_ and 2.0 V_rms_ were used to deform cells (Supplementary File 1). Shear stress during cell deformation was determined from Eqs (1) and (4) with input parameters characterized from experimental measurement for individual cells. It turned out that, under this form of electrical excitation, shear stress in the cell membrane was approximately a constant. Amplitude of the shear stress level was proportional to the excitation voltage. Upon the sudden deactivation of the electric excitation, shear stress in cell membranes returned to zero. The overall shear stress profile during cell deformation was a step-like profile. By inputting such shear stress profile, the nonlinear membrane shear modulus and extensional recovery time, t_c_ into the mathematical model, dynamic deformations in each cell under three voltage levels were determined.

**Figure 4:**
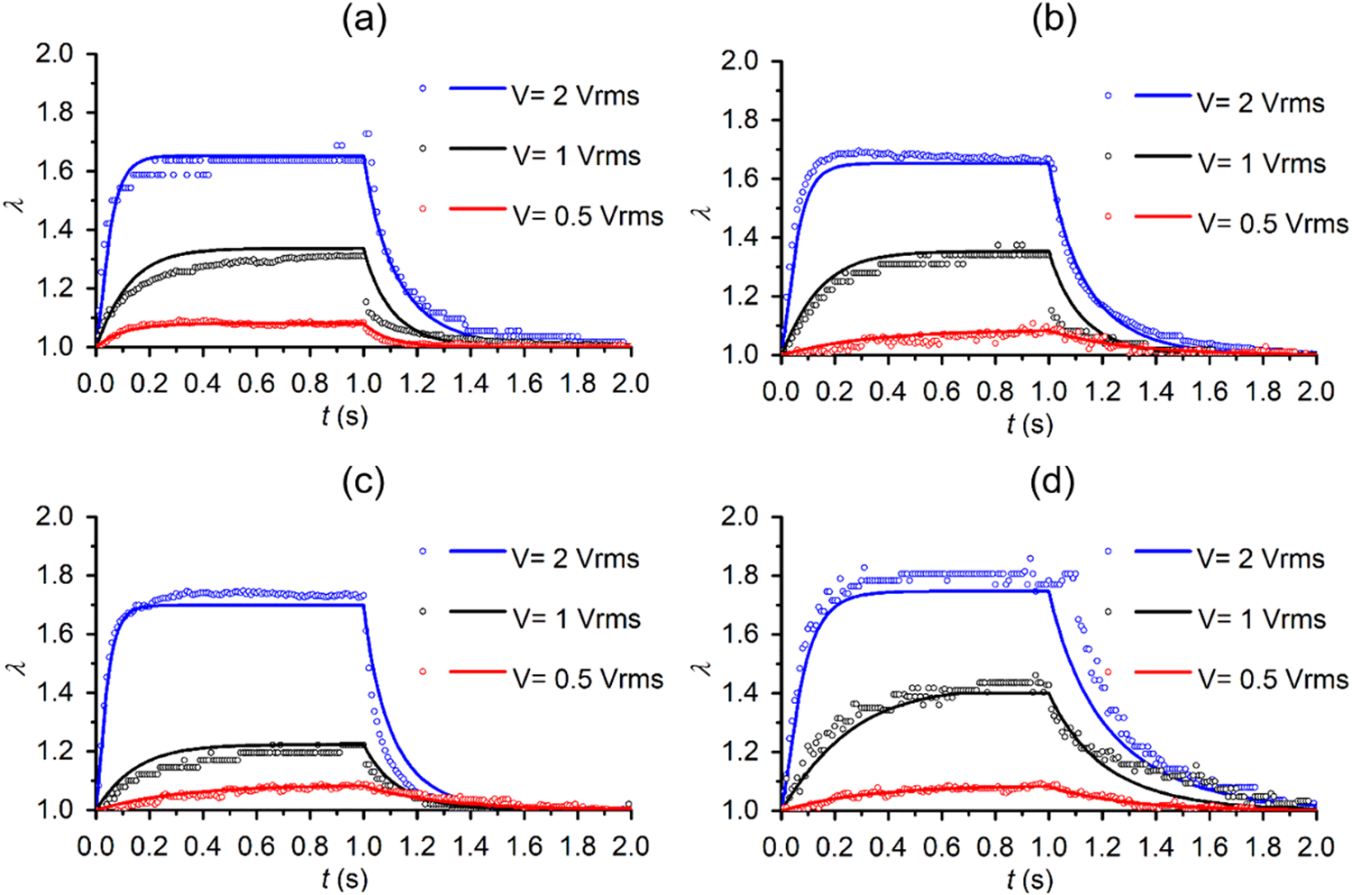
Quantitative comparisons of the theoretical calculations (solid lines) and the experimental observations (open circles) of cell deformation upon electric excitation profile E1 in 4 representative RBCs (**a**–**d**). Excitation voltages 2 V_rms_, 1 V_rms_, and 0.5 V_rms_ are represented by blue, black, and red colors, respectively.

Figure 4 shows that for 4 representative cells, agreement in the cell deformations between the theoretical calculation and the experimental measurements was quantitative. The value of λ increased with the amplitude of the applied voltages. Upon the sudden application of electric excitation, cells were stretched gradually and reached to a plateau in a finite time; upon sudden deactivation of electric excitation, cell membranes returned to the stress-free state. The time required to reach to individual λ plateau decreased with the magnitude of membrane deformation; the time required for cell membranes to relax decreased with the magnitude of the membrane deformation. No plastic deformation was observed in the cells examined in this experiment.

### Cell deformation in response to a stepwise electric excitation

With the validated theoretical model, we further predicted cell deformation in response to a stepwise electric excitation as illustrated schematically by the profile E2 in Fig. 1d. Such excitation profile was generated using an amplitude modulation technique, called amplitude shift keying (ASK), by modulating the high frequency sinusoidal waveform with the stepwise increase and decrease in the amplitude of the electric voltage. The applied voltage was increased from zero to 0.5 V_rms_, 1 V_rms_ and 2 V_rms_ and decreased to zero symmetrically. Duration of each step remained for 1 s and 0.25 s, respectively (Supplementary File 2). In the first case, cell membranes can reach to a plateau before the voltage rises to the next level. In the latter case, the duration at each voltage level is insufficient for cell membranes to reach to an equilibrium deformation state. These two specific cases allowed us to predict the deformations in cell membranes due to the viscoelasticity of cell membranes in response to a specific stress profile.

**Figure 5:**
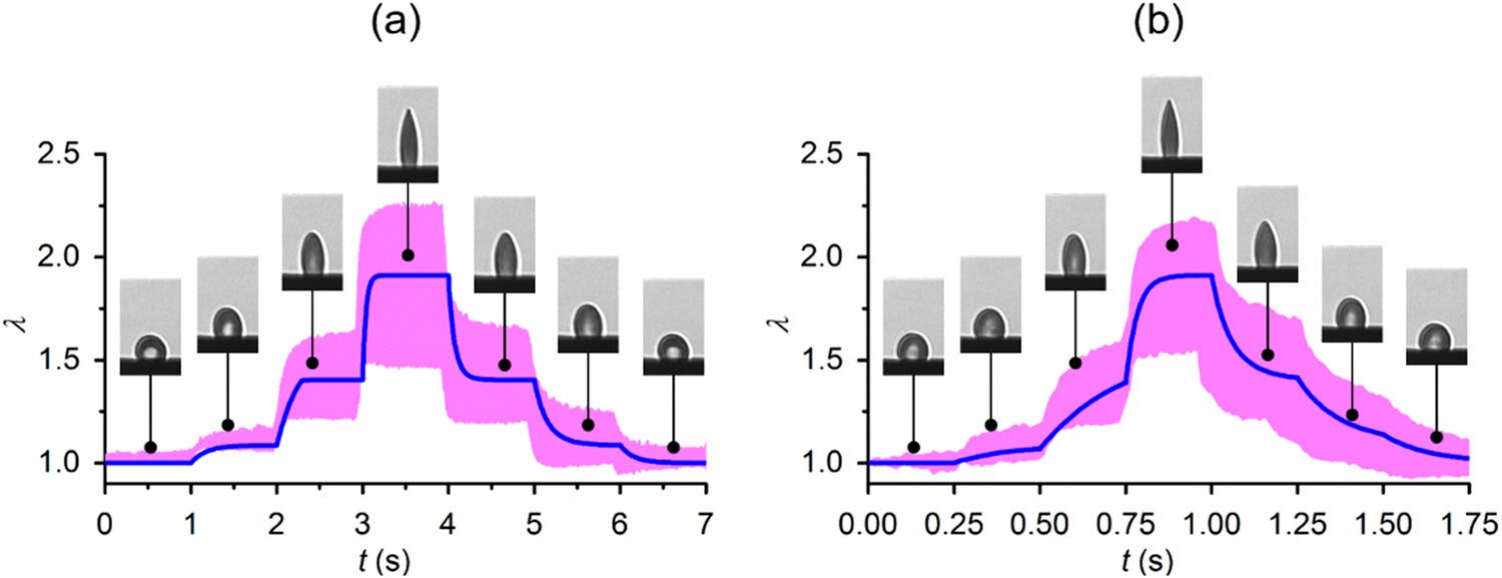
Quantitative comparisons of the theoretical predictions and the experimental observations (n = 10) of cellular deformations in response to a stepwise electric excitation E2. (**a**) Duration of each step is 1 s. (**b**) Duration of each step is 0.25 s. Insets show cell deformations of a representative RBC measured at specific times. The blue lines represent the theoretical calculation. Pink band represent the cell deformation profiles from 10 measurements.

In response to the stepwise excitation, the response of individually tracked RBCs (n = 10) were measured. Deformations of RBCs were predicted based on the theoretical model and compared to the experimental measurements for validation. Good agreement was found between the theoretical calculations and experimental observations for both step durations (Fig. 5a,b). Viscosity of cell membranes determines the time needed to reach to the new equilibrium shape. The response times of the creep deformation decreased with the level of extension ratio. The response times of the relaxation processes were found to be symmetrical to the creep processes. As the step size decreased, the course of membrane deformation became smoother.

### Cell deformation in response to triangular and sinusoidal electric excitations

**Figure 6:**
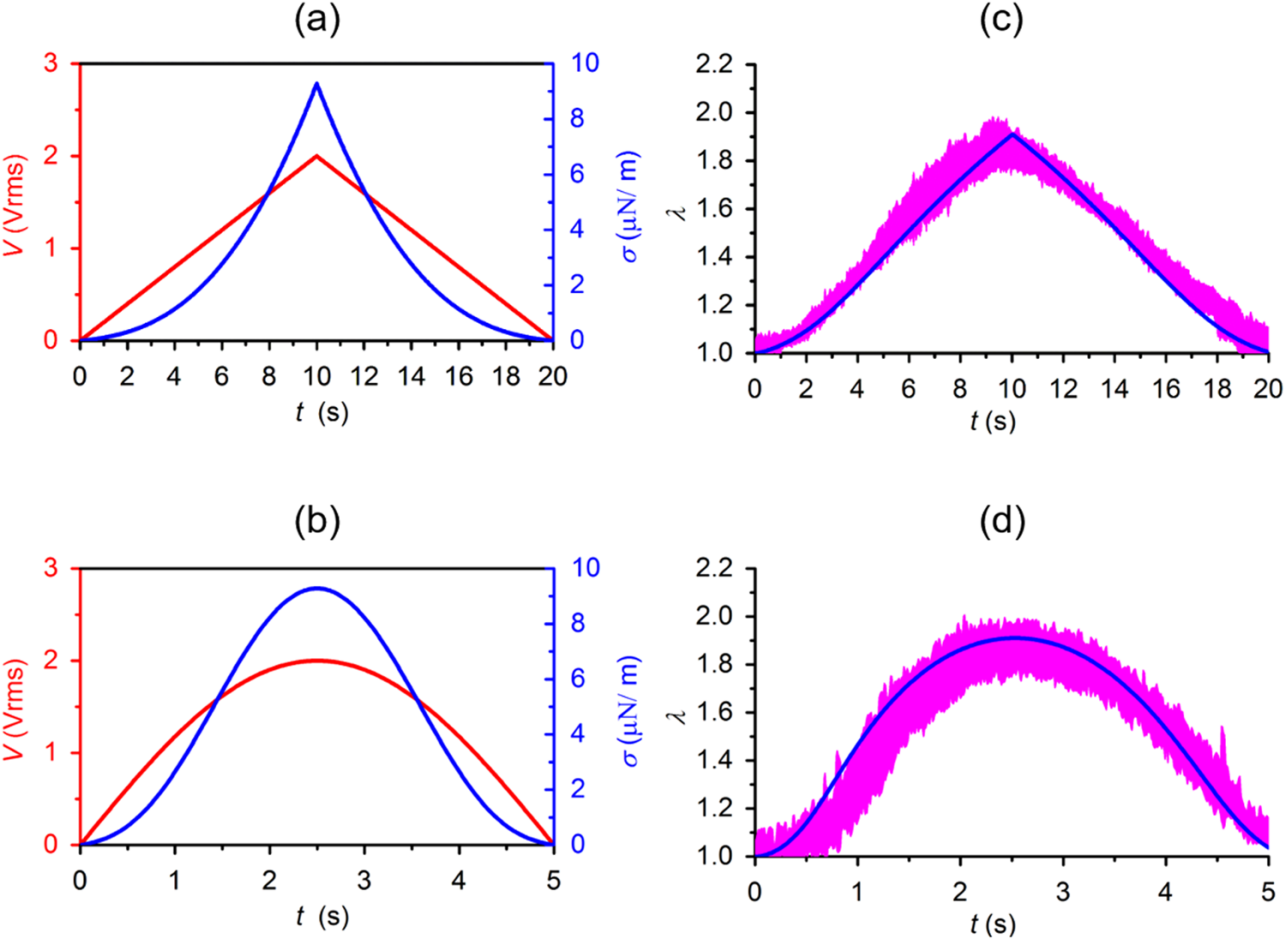
Quantitative comparisons of the theoretical predictions and the experimental observations (n = 10) of cellular deformations in response to: (**a**) a triangular waveform excitation E3 (red curve) and the consequent shear stress (blue curve); and (**b**) a sinusoidal waveform excitation E4 (red curve) and the consequent shear stress (blue curve). (**c**,**d**) Theoretical predictions of cellular deformations (blue curve) and the experimental observations (n = 10, pink band).

The validated theoretical model of DEP-induced cell deformation was then used to predict cellular deformation of a simulated cell in response to a triangular waveform and a sinusoidal waveform modulated by Amplitude Shift Keying (ASK) method as described by E3 and E4 profiles in Fig. 1d (Supplementary File 3). The predictions were compared to experimental observations for validation (Fig. 6). In the first electric excitation, voltage amplitude of the high frequency ac signal was modulated as ${{\rm{V}}}_{{\rm{rms}}}=0.2\times {\rm{t}}({\rm{t}} < 10\,{\rm{s}})$ and ${{\rm{V}}}_{{\rm{rms}}}=4-0.2\times {\rm{t}}\,(10\,{\rm{s}}\le {\rm{t}}\le 20\,{\rm{s}})$, as illustrated by the red line in Fig. 6a. The simulated cell had shear stress values ${\rm{\sigma }}=3.65\times {{{\rm{V}}}_{{\rm{rms}}}}^{2}-2.63\times {{\rm{V}}}_{{\rm{rms}}}+0.71$ and viscoelastic properties $\,{\rm{\mu }}=0.21\times \exp (2.06\times {\rm{\lambda }})-0.25$ averaged from a population of RBCs (n = 84), as shown in Fig. 6a by the blue curve. The second electric excitation was achieved by modulating the high frequency ac signal with voltage level defined as ${V}_{{\rm{rms}}}=2\times \,\sin ({\rm{\pi }}\times {\rm{t}}/5)$, as illustrated by the red line in Fig. 6b. The simulated cell had corresponding shear stress values as illustrated by the blue line. These simulations allowed us to predict the averaged viscoelastic behavior of a population of cells in response to specific waveforms. Quantitative comparison of the theoretical prediction of the extension ratio λ in the simulated cell and the experimental measurements of 10 individually tracked cells validated both simulations (Fig. 6c,d).

Furthermore, we calculated the dissipation energy from the stress-strain hysteresis in the simulated cell in response to the triangular and sinusoidal waveforms, both with peak values of 1 V_rms_ and 2.5 V_rms_. Upon a triangular waveform excitation, the dissipated energy was about 0.03 μJ/m^2^, 0.18 μJ/m^2^, and 0.30 μJ/m^2^ for peak voltages of 1 V_rms_, 2 V_rms_, and 2.5 V_rms_, respectively. Upon a sinusoidal waveform excitation, the dissipated energy was significantly higher, about 0.12 μJ/m^2^, 0.80 μJ/m^2^ and 1.37 μJ/m^2^ for peak voltage values of 1 V_rms_, 2 V_rms_ and 2.5 V_rms_, respectively.

## Discussion

We have developed a comprehensive theoretical-experimental framework for the quantitative, high-throughput study of cell biomechanics combining ASK modulation and DEP in microfluidics. The amplitude-modulated DEP technique allows for full control on the magnitude and the rate of deformations in cell membranes. This gives it more advantages over existing methods and can be further developed to study the effects of strain rate and loading waveform on deformation of biological cells. The mathematical model used in this study allows for prediction of cell deformations under a specific electric waveform, by approximating a simulated cell with averaged properties and its shear stress as an exponential function of the amplitude of the applied voltage. Moreover, the developed framework allows input from the experimentally measured cell deformation to further calibrate the shear stress profile in individual cells, which gives the ability to predict the cell deformations in single cells.

We applied this technique to characterize the nonlinearity in membrane shear moduli in healthy human RBCs. These results are in the range of reported literature values, obtained by other independent studies. Membrane shear modulus for small deformation obtained by other DEP study^41^ was in the lower range of 1.4–2.5 µN/m. The nonlinear shear moduli of RBC membranes determined from an advanced cell model^34^ was found to be 5.3–11.3 µN/m for small deformations, 2.4–5.0 µN/m for large deformations, and 13.9–29.6 µN/m prior to failure. Possible factors contributing to the discrepancy may include variations in the theoretical models, samples, experimental conditions, optical resolution of the measuring systems, and the method for force calibration among different studies. Small variation in the mean recovery characteristic time, t_c_, was found when cells were initially stretched at different voltage levels. Such variation was likely attributed to the variations in the initial shape and dimensions of RBCs among different measurements. Herein, t_c_ was assumed as a constant and averaged as 0.14 ± 0.05 s, which is in consistent with the reported values, 0.19 ± 0.06 s by optical tweezers, and 0.1–0.13 s by micropipette aspiration^36,42^.

Shear stress on cells in this study was generated by the DEP force, which is mainly related to the field strength of the electric excitation. In many DEP studies, geometrical approximations, such as classical spherical model (a = b = c), prolate (a > b = c) and oblate (a = b > c) models, have been used to estimate the force exerted on spherical and non-spherical bioparticles^43^. However, in the case of RBC deformation, spherical model is no long valid; the prolate and oblate assumptions may be valid for large deformations but can still overestimate the DEP force for small deformations. This is because in uniaxially stretched RBCs, b value is usually higher than c value. Therefore, an ellipsoid (a ≠ b ≠ c) can be a suitable model to improve the accuracy of fore calibration. In the direction normal to the bottom wall, cell asymmetry was neglected in the present study and a prior independent study^44^. In the present study, the variation in the c axis was found to be significantly smaller than the observed deformations in the other two axes. In addition, we found that the calibrated DEP force is weakly dependent on the deformation in the c axis. When the surface area is considered to be globally conserved, a decrease of 0–5% in the calibrated DEP force was observed for 0.5. V_rms_ to 2 V_rms_. In the stretching direction, cell symmetry was verified by replacing gold electrode with transparent indium tin oxide electrode^26^. Thus, in present study, the part of cell view unblocked by the gold electrodes was utilized and fitted with ellipse to quantify cell deformation. It should also be noted that even though the triaxial ellipsoid model may be very close to an uniaxially deformed RBC, it can still overestimate the DEP force to a certain extent, as tip formation occurred in RBCs undergoing large deformations^19^. Consequently, the characterized shear modulus for large deformations may be slightly higher than the true values. Numerical simulations using finite element model, such as MST have been proposed to improve the accuracy of the calibrated DEP force. Comparing to the MST method, EDM method overestimated the DEP forces, which also explains why the characterized value of shear modulus are higher than the previous work^40^.

We found that the energy dissipation calculated from the stress-strain hysteresis loop is closely related to the magnitude, type and frequency of the applied waveforms. The value of dissipated energy may provide a more general criterion than cell deformation for quantitative comparison among different studies. For instance, in our prior work^32^, RBC membrane failure occurred after approximately 180 cycles of DEP-based stretching and relaxation at a field strength of 1 kV/cm for 10 s pulse duration. A similar DEP work^45^ showed that RBC fatigue failure started after 300 cycles of 0.1 s pulse duration of 4 kV/cm field strength. Another work^46^ using passive microfluidics method, found that RBCs fatigue failure appeared after 500 cycles of reciprocated mechanical stress in a microchannel. Such significant difference in the reported results is mainly due to variations in loading profiles and experimental methods. In this case, an energy-based fatigue life prediction method, which has been well developed for structural materials^47,48^ may provide a quantitative comparison among different fatigue studies of biological cells. On the other hand, cell membrane deformation and shear stress are coupled with cellular electrical properties, as the DEP force exerted on cell membranes is strongly dependent on the polarizability of the cell membrane and cytoplasm. Considering RBCs affected by diseases, such as malaria and sickle cell disease, cells may have significantly different electrical properties. Therefore, even under a same field strength, the magnitude of the DEP force and the shear stress may be different between normal and diseased cells. The validated theoretical model in this study may provide a new opportunity to quantitatively compare the energy dissipation of viscoelastic biological cells in both health and disease.

## Conclusions

This study presented a novel electrodeformation technique using ASK modulated DEP force in a microfabricated platform as well as a validated theoretical modeling of cell electrodeformation. Systematic experiments of the stretching and relaxation responses of RBCs subjected to variant loading waveforms were performed using this developed technique. The mathematical models utilized an ellipsoid multi-shell model and the MST method for an accurate calibration of DEP forces. Electrodeformation prediction from the mathematical models matched well the experimental results in extension ratio for uniaxial stretching and recovery in single RBCs and in cell populations. Specifically, deformations of cell membranes at constant and variant loading rates were achieved using a triangular loading waveform and a sinusoidal loading waveform, which are envisioned as a useful fundamental work of cell biomechanics and can be extended to study cellular fatigue life based on the theory of energy dissipation.

## Methods

### Ethics statement

All the experimental methods were carried out in accordance with the approved guidelines. The blood collection procedure was verified and approved by the Institutional Review Board (IRB) of Florida Atlantic University. Informed consent was obtained from the sample donor.

### Experiment setup

The microfluidic device consisted of a 50 μm deep polydimethylsiloxane (PDMS) microchannel and a 0.7 mm thick glass chip, permanently bonded using air plasma (Fig. 1a). The glass substrate was coated with an interdigitated thin-film electrode array (IEA) of Ti (10 nm)/Au (100 nm) film patterned using standard microfabrication techniques^23^. The IEA structure consisted of 20 µm gap and 20 µm band width. Different DEP loading profiles were achieved by adjusting the voltage of a sinusoidal waveform through the IEA using a signal generator (SIGLENT SDG830, SIGLENT, China) (Fig. 1d). The electric frequency was set at 1.58 MHz, which gave a favorable positive DEP force to deform cell membranes in the current setup (Supplementary Fig. S1). RBC deformation was visualized and recorded at 100 frames per second via a high-resolution GigE Camera (The Imaging Source, Charlotte, NC) mounted on an Olympus X81 inverted microscope. A 414 ± 46 nm band pass filter was inserted in the optical path for improved visualization, as this wavelength is near the peak of the hemoglobin absorption spectra^49^.

### Sample preparation

Fresh blood specimen was obtained by finger pricking from a healthy donor, and washed twice with phosphate-buffered saline (PBS, Lonza Walkersville, Inc., Walkersville, MD) at 2000 rpm for 2 min at room temperature. Isotonic working buffer containing 8.5% (w/v) sucrose and 0.3% (w/v) dextrose was prepared. Its electrical conductivity was adjusted to 0.018 S/m using PBS. The RBC pellet was collected and diluted to 10^6^ cells/ml in the working buffer. Before each test, the microfluidic channel was coated with the working buffer containing 5% bovine serum albumin (BSA, Lot 20150520AS, Rocky Mountain Biologicals. Inc, Missoula, MT) for 30 min to prevent cell adhesion to the bottom of the channel. Excess BSA in the channel was removed with the working buffer before cells were loaded into the device. Testing was performed at room temperature.

### DEP force calibration

DEP-induced electrodeformation offers an effective and relatively high throughput means to characterize the biomechanics of single cells^23^. Under a positive DEP condition, deformable biological cells, such as RBCs, can be firmly trapped at the electrode edges and elongated by the repelling force at one of the induced dipole away from the electrode edges (Fig. 1c), due to the interfacial Maxwell–Wagner polarization across cellular membranes^50^. DEP forces in response to different levels of field strength were calibrated by and compared between two methods: the EDM method based on an improved mathematical model and the MST method.

Assuming a stretched RBC as an ellipsoid, the time-averaged DEP force can then be quantified by^21^

1 $$\langle {F}_{DEP}\rangle =2\pi abc\cdot {\varepsilon }_{m}\cdot Re({f}_{CM})\cdot \nabla {E}_{rms}^{2}$$

where a and b are the radii along x and y axes of the fitted ellipse of the cell shape, extracted from the recorded image sequences using a custom, MATLAB script (Fig. 1b). Radius along the z axis of the deformed RBC, c is small and nearly constant during deformation^44,51^, and assumed as a constant, 1.3 µm^44,52^. *ε* is the permittivity of the surrounding medium, and ∇*E* is the root-mean-square value of the gradient of electric field strength square, proportional to the magnitude of the DEP force field (Fig. 1e), simulated by COMSOL Multiphysics 5.2 (COMSOL, Inc., Burlington, MA). Value of ∇*E* is related to the amplitude of voltage and the distance d between the center of cell and the edge of the electrode (Supplementary Fig. S2). *Re*(*f*_*CM*_) is the real part of the Clausius Mossotti factor (*f*_*CM*_), which is calculated using a custom MATLAB script^32^. As a RBC consists of membrane and cytoplasm, its effective permittivity can be estimated with a single-shell structure, following a concentric multi-shell mode^51,53^,

2 $${f}_{CM}=\frac{1}{3}\frac{({\varepsilon }_{mem}^{\ast }-{\varepsilon }_{m}^{\ast })[{\varepsilon }_{mem}^{\ast }+{A}_{1}({\varepsilon }_{cyto}^{\ast }-{\varepsilon }_{mem}^{\ast })]+\rho ({\varepsilon }_{cyto}^{\ast }-{\varepsilon }_{mem}^{\ast })[{\varepsilon }_{mem}^{\ast }-{A}_{1}({\varepsilon }_{mem}^{\ast }-{\varepsilon }_{m}^{\ast })]}{({\varepsilon }_{m}^{\ast }+{A}_{1}({\varepsilon }_{mem}^{\ast }-{\varepsilon }_{m}^{\ast }))[{\varepsilon }_{mem}^{\ast }+{A}_{1}({\varepsilon }_{cyto}^{\ast }-{\varepsilon }_{mem}^{\ast })]+\rho {A}_{2}(1-{A}_{2})({\varepsilon }_{cyto}^{\ast }-{\varepsilon }_{mem}^{\ast })({\varepsilon }_{mem}^{\ast }-{\varepsilon }_{m}^{\ast })}$$

where the subscripts cyto, mem and m stand for cytoplasm, membrane and medium, respectively.$${{\rm{\varepsilon }}}^{\ast }={\rm{\varepsilon }}-j{\rm{\sigma }}/{\rm{\omega }}$$ with ω, ε and σ as the angular frequency, dielectric permittivity and conductivity, respectively. $$\rho =(a-t)$$$$(b-t)(c-t)/(abc)$$. Particularly, *ε*_mem_ = 4.44, *ε*_cyto_ = 59, *σ*_mem_ = 10 *S*/*m*, *σ*_cyto_ = 0.31 *S*/*m*, adopted from other publication^54^. $${A}_{i=1,2}\,\,$$is the depolarization factor, defined as

3 $${A}_{i}=\frac{{a}_{i}{b}_{i}{c}_{i}}{2}{\int }_{0}^{\infty }\frac{{d}_{s}}{(s+{a}_{i}^{2}){B}_{i}},\,i=1,\,2$$

where ${B}_{i}=\sqrt{((s+{a}_{i}^{2})(s+{b}_{i}^{2})(s+{c}_{i}^{2}))}$, ${a}_{1}=a,\,{b}_{1}=b,{c}_{1}=c$, ${a}_{2}=a-t,{b}_{2}=b-t,{c}_{2}=c-t$, and t denotes the thickness of cell membrane, 4.5 nm.

Alternatively, the DEP force can be calculated by integrating the Maxwell stress tensor over the surface of the cell volume to yield the electric fields. The time-averaged tensor is given by^55^.

4 $$\langle {{\boldsymbol{\sigma }}}^{{\rm{MST}}}\rangle =\frac{1}{4\,}Re[\tilde{\varepsilon }]({\boldsymbol{EE}}^{\prime} +{\boldsymbol{E}}^{\prime} {\boldsymbol{E}}-{|{\boldsymbol{E}}|}^{2}{\boldsymbol{I}})$$

where $\tilde{\varepsilon }$ is the complex electrical permittivity, ***E*** the electrical field, ***I*** is the unit tensor, and the product of two vectors denotes the dyadic product. The deformed cell is also modelled as a triaxial ellipsoid shape with the same dimensions as measured in the EDM method. Hence, the time averaged DEP force exert on the cell can be obtained by the following integration

5 $$\langle {{\boldsymbol{F}}}_{DEP}\rangle =\underset{S}{\overset{\,}{\oint }}\langle {{\boldsymbol{\sigma }}}^{{\rm{MST}}}\rangle \cdot {\boldsymbol{n}}dS$$

where $S$ is the outer surface area of the cell model, and ***n*** is the unit vector normal to the cell surface. These integrations were conducted numerically in the finite element analysis package COMSOL Multiphysics as mentioned above (Fig. 1f).

### Theoretical model for viscoelastic behavior of RBC membranes

In response to DEP actuation, RBC exhibits a time-dependent deformation due to its inherent viscoelasticity of cell membrane. To model such viscoelastic behavior, a time-dependent constitutive Kelvin-Voigt solid model^30,36^ can be used (Fig. 1g). Specifically, shear stress σ from the time-variant DEP loading is determined by averaging half of the DEP force in the stretching direction over the minor axis 2b,

6 $${\rm{\sigma }}({\rm{t}})=\frac{{F}_{DEP}({\rm{t}})}{4{\rm{b}}({\rm{t}})}\,$$

The induced shear strain ε is calculated by

7 $${\rm{\varepsilon }}({\rm{t}})=\,\frac{({\rm{\lambda }}{({\rm{t}})}^{2}-{\rm{\lambda }}{({\rm{t}})}^{-2})}{2}\,$$

where b is the minor semi-axis, ${\rm{\lambda }}({\rm{t}})={{\rm{b}}}_{0}/{\rm{b}}({\rm{t}})\,\,$is the extension ratio of the initial minor semi-axis b_0_ over the transient minor semi-axis b(t). Then the transient deformation of single RBCs can be described by

8 $$\frac{{\rm{\sigma }}({\rm{t}})}{2\,{\rm{\mu }}({\rm{\lambda }})}=\frac{1}{4}({\rm{\lambda }}{({\rm{t}})}^{2}-{\rm{\lambda }}{({\rm{t}})}^{-2})+{{\rm{t}}}_{{\rm{c}}}\frac{\partial \,\mathrm{ln}\,{\rm{\lambda }}({\rm{t}})}{\partial {\rm{t}}}$$

where ${\rm{\mu }}$ is membrane shear modulus, ${{\rm{t}}}_{{\rm{c}}}\equiv {\rm{\eta }}/{\rm{\mu }}$ is the recovery characteristic time, and ${\rm{\eta }}$ is membrane viscosity. Value of ${{\rm{t}}}_{{\rm{c}}}$ can be extracted by an exponential fit of the experimental measurements of stretch ratio (SR = a/b) at σ = 0,

9 $$\exp (-\frac{{\rm{t}}}{{{\rm{t}}}_{{\rm{c}}}})=\frac{({\rm{SR}}-{{\rm{SR}}}_{\infty })({{\rm{SR}}}_{0}+{{\rm{SR}}}_{\infty })\,}{({\rm{SR}}+{{\rm{SR}}}_{\infty })({{\rm{SR}}}_{0}-{{\rm{SR}}}_{\infty })}\,$$

where SR_0_ and SR_∞_ represent the cellular SR measured at the point of DEP release and when cell membrane recovers to its stress-free state, respectively.

## Electronic supplementary material

Dataset 1

Supplementary Figures
