# Supplementary material for: Modeling erythrocyte electrodeformation in response to amplitude modulated electric waveforms
Source: Sci Rep. 2018 Jul 5;8:10224. doi: 10.1038/s41598-018-28503-w (PMC6033869; doi:10.1038/s41598-018-28503-w)
Supplement: Supplementary file 2 — Supplementary Figures [file 41598_2018_28503_MOESM2_ESM.pdf]

Modeling erythrocyte electrodeformation in response to amplitude modulated electric waveforms

Yuhao Qiang [1], Jia Liu[1], Fan Yang[2], Darryl Dieujuste[3] and E Du[1,*]

[1]Department of Ocean and Mechanical Engineering, Florida Atlantic University, Boca Raton, FL 33431, USA.
[2]School of Mechanical Engineering, Nanjing University of Science and Technology, Nanjing, 210094, CHN.
[3]Department of Electrical Engineering, Florida Atlantic University, Boca Raton, FL 33431, USA.

* Correspondence: edu@fau.edu

Supplementary figures

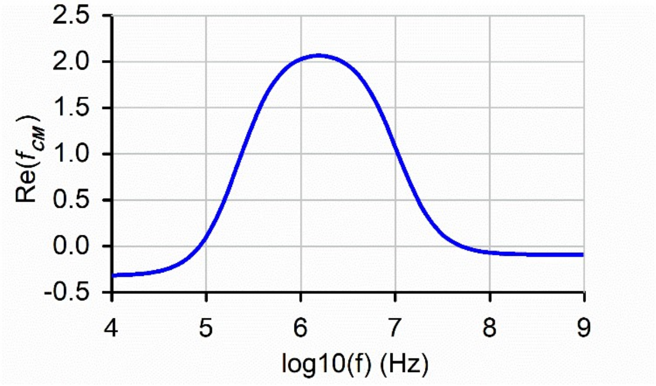

Figure S1. Profile of Re(f_{CM}) of a healthy human red blood cell determined based on a single-shell ellipsoidal model.

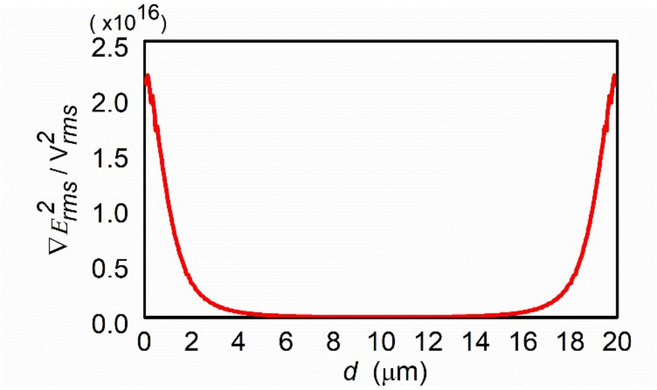

Figure S2. Value of $\nabla^2 E_{\text{rms}}/\text{V}^2_{\text{rms}}$ measured as a function of distance, d between the center of cell and the edges of the electrode (d = 0 μm and d = 20 μm) from COMSOL Multiphysics simulation.
